# Ingested toothpick retrieved through a lumbar approach: a case report

**DOI:** 10.1186/s12893-020-00768-x

**Published:** 2020-05-12

**Authors:** Chun-Run Ling, Yi Chen, Chun-Gang He

**Affiliations:** 1 Department of General and Pediatric Surgery, The People’s Hospital of Guangxi Zhuang Autonomous Region, 6 Tao Yuan Road, Nanning, 530021 Guangxi Zhuang Autonomous Region China; 2 Division of Colorectal & Anal Surgery, Department of Gastrointestinal Surgery, Guangxi Medical University Cancer Hospital, Nanning, Guangxi Zhuang Autonomous Region China

**Keywords:** Ingested foreign body, Toothpick, Gastrointestinal perforation, Local anesthesia, Case report

## Abstract

**Background:**

Ingested toothpick may cause severe complications if there is no intervention timely. Toothpicks that required surgical intervention often retrieved through exploratory laparotomy or laparoscopic exploration surgery under general anesthesia, while, those through lumbar approach have been rarely reported. Herein, authors report a case of ingested toothpick which removed through the lumbar surgical approach under local anesthesia and the patient has gained a considerable recovery.

**Case presentation:**

A 57-year-old man was admitted to our hospital with distending pain in the right flank for more than 20 days. He had a history of accidental toothpick ingestion. Abdominal computed tomography (CT) scan and Color Doppler Ultrasound of the superficial tissue (right flank pain area) consistently revealed a linear lesion -corresponding to the toothpick- was located at the right flank next to the body surface. Surgery via lumbar approach was then successfully performed to retrieve the toothpick under local anesthesia. The post-procedural course was uneventful, and the patient was discharged on the third day after surgery, no complications were noted at the 18-month follow-up.

**Conclusion:**

When a foreign body that causes perforation of the digestive tract remains for a relative long time (non-acute stage) and the perforation is close to the body surface, a local anesthesia surgery through the corresponding body surface may be a considerable choice.

## Background

Digestive tract perforation is one of the most common complications of ingested foreign bodies [1]. Generally, foreign bodies that cause perforations in the gastrointestinal tract and require surgical intervention are usually retrieved through exploratory laparotomy or laparoscopic exploration surgery. In contrast, removal of foreign bodies using the lumbar approach are rarely reported. Here, we report the case wherein a toothpick was retrieved using the lumbar surgical approach under local anesthesia.

## Case presentation

**Fig. 1:**
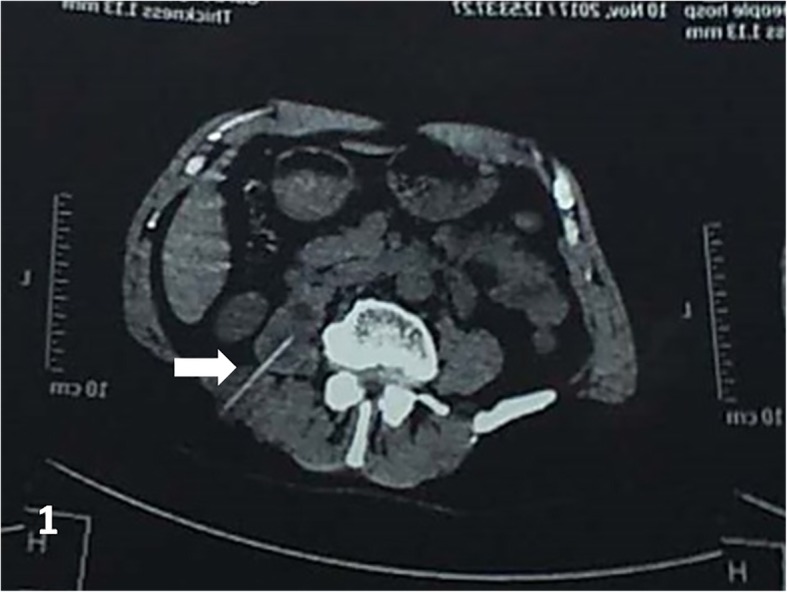
Computed tomography of the abdomen revealed a hyperdense foreign body traversed across the right psoas and sacrospinalis muscle (arrow)

**Fig. 2:**
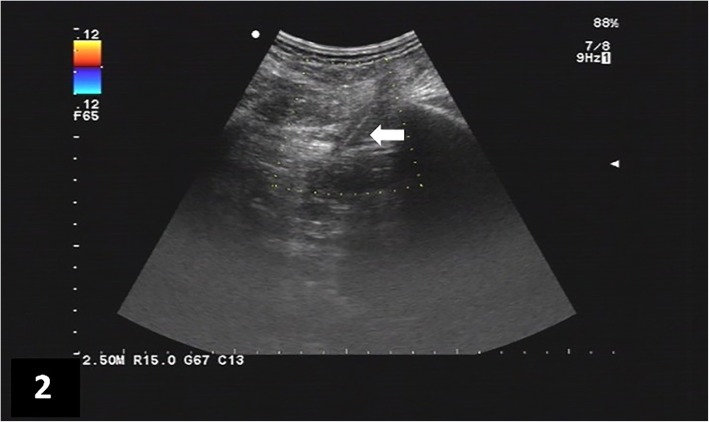
A hyperechoic linear lesion located in a low echo areal between the subcutaneous fat layer and intramuscular layer of the right flank was showed in color Doppler ultrasound image (arrow)

**Fig. 3:**
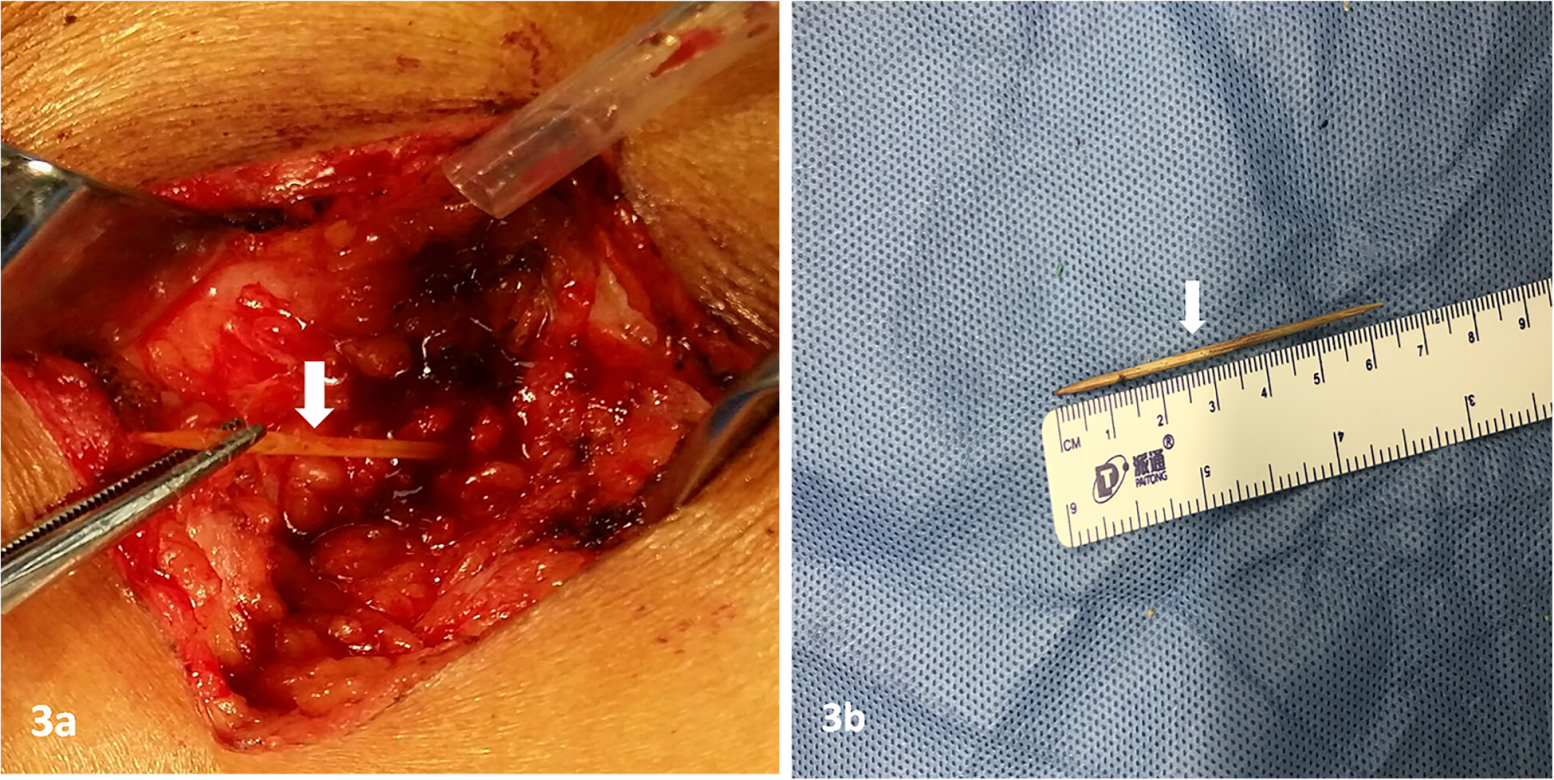
**a** and **b**. The toothpick, 63 mm in length, was found during the operation (arrow)

A 57-year-old man was admitted to our hospital with distending pain in the right flank for more than 20 days. He swallowed a toothpick by accident in the days immediately prior to symptom onset. Since there were no abdominal symptoms or signs that would interfere with normal eating, he did not try to trace a toothpick excretion. He did not complain of abdominal radiating pain; he also did not have any other complaints. For the pain in the right flank, he received treatment of anti-infection and analgesics at a different institution (details unknown) before admission to our hospital; however, his symptoms improved only slightly. Physical examination results were, as follows: Temperature 36.5 °C, Pulse rate 76/min, Respiratory rate 20/min, and Blood pressure 132/71 mmHg, i.e., his vital signs were stable. The abdomen was soft, without tenderness or rebound tenderness, muscle tension, or abnormal mass. The fluid thrill test and shifting dullness sign were negative, and bowel sound frequency was 3/min. Tenderness and percussion pain were observed in the right flank, without redness, swelling, or increased skin temperature in this area, the fluctuation test was normal. The abdominal CT scan revealed a residual foreign body traversed across the right psoas and sacrospinalis muscle, accompanied with inflammation of the surrounding soft tissues (local abscess formation could not be ruled out; Fig. 1). Color Doppler ultrasound of the superficial tissue (right flank pain area) showed that a hyperechoic linear lesion, i.e., the toothpick, was located in a low echo areal between the subcutaneous fat layer and intramuscular layer of the right flank (Fig. 2). Abdominal plan X-ray graphy showed no subdiaphragmatic free air, and the routine blood tests and serum C-reactive protein (CRP) levels were within the normal range. With the foreign body located by ultrasound performed in the prone position, the patient was then operated on under local anesthesia (local infiltration anesthesia with 0.5% lidocaine was administered in operative site instantly before operation). The skin and subcutaneous tissues were incised sharply, and a horizontal incision was made in the right lumbar region. The local soft tissue of this area presented with chronic inflammatory change. After separation of the inflammatory granulation tissue, the sharp point of the toothpick was found on the surface of the right sacrospinalis muscle. The toothpick was then removed (Fig. 3 a and b). After the inflammatory granulation tissue was fully scraped out, the surgical area was washed with hydrogen peroxide and saline, and a drainage tube was inserted at the incision site. The subcutaneous tissue and skin were sutured layer by layer. The operation took 32 min. Piperacillin and tazobactam injection was used as an antibiotherapy after surgery. The post-procedural course was uneventful. On the third day after the operation, the patient’s blood routine was normal and there was no fluid drawn from the drainage tube, the patient was then discharged after the drainage tube was removed. No complications were noted at the 18-month follow-up.

## Discussion and conclusion

Ingested foreign bodies are not rare in clinical practice, about 10% ~ 30% of them occurred in the esophagus, 80% of the foreign bodies reaching the stomach could pass spontaneously, only 12–16% required surgical intervention [2]. Nearly 20% of the patients with gastrointestinal foreign bodies would develop complications, such as perforation, hemorrhage, obstruction, foreign body fixation, etc., which often occur in the physiological curvature or stenosis of the digestive tract. Toothpicks, usually present 6 cm in length with sharpened ends, making perforation a common complication. Secondary damage to the liver, pancreas, kidney, or heart might happen at corresponding perforation site [3]. According to the *Management of ingested foreign bodies and food impactions* which recommended by the American Society for Gastrointestinal Endoscopy (ASGE) in 2011 [4], sharp foreign bodies reaching the stomach or proximal duodenum should be retrieved endoscopically if possible, and surgical intervention should be considered for objects that fail to progress within 3 days confirmed by a daily-radiograph. In previous reports, toothpicks that required surgical intervention often retrieved through exploratory laparotomy or laparoscopic exploration surgery under general anesthesia [5, 6]. To our best knowledge, this is the first case that removing an ingested toothpick through a lumbar surgical approach under local anesthesia, by which the patient gained less trauma, less use of analgesics, less hospital stay and less hospital expenses. In our case, the patient had a course up to more than 20 days, physical examination did not show the signs of peritonitis, and the relevant auxiliary examination suggested that the toothpick had been fixed with one of its ends close to the right flank surface, the inflammatory indicators suggested a non-inflammatory acute phase, indicating that perforation has been limited. Severe locally inflammatory edema, adhesion, or fibrous wrappage might occur at the area of perforation simultaneously [6, 7], these make an abdominal surgery (laparotomy or laparoscopic surgery) rather difficult. Sometimes an intestinal resection surgery should be arranged followed by the removal of the foreign bodies [5], resulting in a relatively large surgical trauma and potential abdominal contamination. According to the literatures [6, 8], it is safe to remove a pierced toothpick in digestive tract with a chronic cause as the inflammation is limited, which is confirmed in our case. Even so, the passage of the toothpick might cause chronic inflammation of the intestinal wall, mucosal hyperplasia and even canceration. Periodic reexamination of electronic colonoscopy after surgery is a necessary means for early detection of these lesions. However, the patient refused further examination because there was no discomfort after surgery. We will continue to follow him up closely. If he has gastrointestinal symptoms such as abdominal pain, change of stool habits, and bloody stool, etc., we will urge him to return to the hospital for further reexamination. We suggest that a thorough history, combined with necessary assist examinations such as endoscopy, ultrasound and CT might help to identify the perforation site of a pierced toothpick and gain an optimal treatment decision.

In conclusion, this case demonstrates that not all digestive tract foreign bodies that require surgical intervention need to be operated through a laparotomy or laparoscopic surgery, when a sharp foreign body remains for a relative long time (non-acute stage) and the perforation is close to the body surface, it is safe and less traumatic to remove the foreign body by a local anesthesia surgery through the corresponding body surface.

## Data Availability

The datasets used and/or analysed during the current study are available from the corresponding author on reasonable request.

## Abbreviation

CT: Computed Tomography
